# Identification of FCN1 as a novel macrophage infiltration-associated biomarker for diagnosis of pediatric inflammatory bowel diseases

**DOI:** 10.1186/s12967-023-04038-1

**Published:** 2023-03-17

**Authors:** Xixi Chen, Yuanqi Gao, Jinfang Xie, Huiying Hua, Chun Pan, Jiebin Huang, Mengxia Jing, Xuehua Chen, Chundi Xu, Yujing Gao, Pu Li

**Affiliations:** 1 Department of Pediatrics, Ruijin Hospital, Shanghai Jiao Tong University School of Medicine, Ruijin Er Rd.197, Shanghai, 200025 China; 2 NHC Key Laboratory of Metabolic Cardiovascular Diseases Research, Department of Biochemistry and Molecular Biology, School of Basic Medical Sciences, Ningxia Medical University, Yinchuan, 750004 China

**Keywords:** Pediatric inflammatory bowel disease, Diagnostic biomarkers, FCN1, Macrophages, Inflammation

## Abstract

**Background:**

The incidence of pediatric inflammatory bowel disease (PIBD) has been steadily increasing globally. Delayed diagnosis of PIBD increases the risk of complications and contributes to growth retardation. To improve long-term outcomes, there is a pressing need to identify novel markers for early diagnosis of PIBD.

**Methods:**

The candidate biomarkers for PIBD were identified from the GSE117993 dataset by two machine learning algorithms, namely LASSO and mSVM-RFE, and externally validated in the GSE126124 dataset and our PIBD cohort. The role of ficolin-1 (FCN1) in PIBD and its association with macrophage infiltration was investigated using the CIBERSORT method and enrichment analysis of the single-cell dataset GSE121380, and further validated using immunoblotting, qRT-PCR, and immunostaining in colon biopsies from PIBD patients, a juvenile murine DSS-induced colitis model, and THP-1-derived macrophages.

**Results:**

FCN1 showed great diagnostic performance for PIBD in an independent clinical cohort with the AUC of 0.986. FCN1 expression was upregulated in both colorectal biopsies and blood samples from PIBD patients. Functionally, FCN1 was associated with immune-related processes in the colonic mucosa of PIBD patients, and correlated with increased proinflammatory M1 macrophage infiltration. Furthermore, single-cell transcriptome analysis and immunostaining revealed that FCN1 was almost exclusively expressed in macrophages infiltrating the colonic mucosa of PIBD patients, and these FCN1^+^ macrophages were related to hyper-inflammation. Notably, proinflammatory M1 macrophages derived from THP-1 expressed high levels of FCN1 and IL-1β, and FCN1 overexpression in THP-1-derived macrophages strongly promoted LPS-induced activation of the proinflammatory cytokine IL-1β via the NLRP3-caspase-1 axis.

**Conclusions:**

FCN1 is a novel and promising diagnostic biomarker for PIBD. FCN1^+^ macrophages enriched in the colonic mucosa of PIBD exhibit proinflammatory phenotypes, and FCN1 promotes IL-1β maturation in macrophages via the NLRP3-caspase-1 axis.

**Supplementary Information:**

The online version contains supplementary material available at 10.1186/s12967-023-04038-1.

## Background

Inflammatory bowel diseases (IBD), including Crohn’s disease and ulcerative colitis, are characterized by chronic immune-mediated disorders of the gastrointestinal tract. The incidence of IBD in pediatric populations is steadily increasing worldwide [1, 2]. Pediatric IBD (PIBD) often has an aggressive course and extensive disease distribution [3], and affects the growth and development of children [4, 5], which is different from adult IBD [6]. The clinical presentation of PIBD patients is variable, non-specific, and sometimes non-classical [5, 7], and therefore delays in diagnosis of PIBD are common [8]. Early diagnosis of PIBD is associated with improved long-term outcomes [9, 10]. An increasing number of biomarkers have been investigated to diagnose IBD [11], but there has been limited success in using these diagnostic markers in clinical practice. Fecal calprotectin is the most documented non-invasive marker for IBD [12, 13]. The use of fecal calprotectin in addition to symptoms improves the diagnostic accuracy of PIBD [14], but some PIBD patients have normal fecal calprotectin levels [15, 16]. Therefore, it is still necessary to identify novel useful markers for early diagnosis of PIBD.

Ficolins (FCNs) function as soluble pattern recognition molecules in the first line of host defense by sensing microorganisms and triggering complement activation via the lectin pathway. Three ficolins (FCN1, FCN2 and FCN3) have been characterized in humans, whereas only two ficolins (ficolin-A and ficolin-B) have been identified in mice [17]. Mouse ficolin-B (FCNB) is the homologue of human ficolin-1 (FCN1) [18]. Notably, FCN1 could bind to self-associated sialic acids, which may contribute to the development of autoimmunity [17, 19]. Accumulating evidence indicates the important role of ficolins in autoimmune diseases [20]. A previous study reported that FCN1 mRNA expression was upregulated in peripheral blood mononuclear cells (PBMCs) from adult IBD patients [21]. However, it is not yet fully elucidated the expression level of FCN1 in colorectal mucosa and peripheral blood of PIBD patients, and the underlying role of FCN1 in PIBD.

Intestinal mucosal immune cells including macrophages [22], dendritic cells [23], neutrophils [24], T cells [25] and B cells [26], are involved in the pathogenesis of IBD. However, a comprehensive large-sample analysis of colorectal immune cell infiltration in PIBD is still lacking, and the association between FCN1 expression and immune cell infiltration in PIBD mucosa remains elusive.

In this study, we identified FCN1 as a novel promising mucosal and circulating biomarker for PIBD diagnosis by performing machine learning-based biomarker screening analyses and external validation. We also found that FCN1 was upregulated and involved in immune-related processes in PIBD, and the correlation between FCN1 expression and proinflammatory macrophage infiltration in two bulk transcriptomic datasets and a single-cell dataset [27–29]. Key findings from the bioinformatics analyses were further corroborated in our PIBD clinical cohort and juvenile murine DSS-induced colitis model. Furthermore, we investigated the expression levels of FCN1 in macrophages of different phenotypes and the role of FCN1 in the IL-1β maturation by using THP-1-derived macrophages.

## Methods

### Identification of differentially expressed genes

RNA-sequencing (RNA-seq) data of rectal biopsies from PIBD and non-IBD pediatric patients was downloaded from Gene Expression Omnibus (GEO) (https://www.ncbi.nlm.nih.gov/geo/) under the accession number, GSE117993 [27]. Then, we followed the standard workflow of the ‘DESeq2’ package [30] in R software (version 4.0.4) to identify mucosal gene expression changes between 55 non-IBD controls and 75 PIBD patients. Differentially expressed genes were identified with adjusted *P* values less than 0.05 and Log2FoldChange absolute values greater than 2.

### Potential biomarker discovery and validation

To identify the potential diagnostic biomarkers of PIBD among the differentially expressed genes, we implemented two machine learning algorithms, least absolute shrinkage and select options (LASSO) [31] logistic regression and multiple support vector machine-recursive feature elimination (mSVM-RFE) [32]. We first used the R package ‘glmnet’ (v4.1-1) [33] in for the LASSO logistic regression. Then, mSVM-RFE algorithm was performed by using the R package ‘e1071’ (v1.7-7) and the R implementation of this method available on GitHub (https://github.com/johncolby/SVM-RFE). Subsequently, the top-ranked biomarkers identified by the above two algorithms were entered into logistic regression models. After that, the area under the ROC curve (AUC) was employed to assess the performance of these models based on the external validation dataset (GSE126124) [28]. The GSE126124 dataset consisted of gene expression microarray data of blood and colon samples from 59 PIBD patients and 39 non-IBD children, so we divided this dataset into two parts (blood and tissue) for validation.

### Functional enrichment analysis

Differentially expressed genes identified above were subjected to Gene Ontology (GO) enrichment analysis using the R package ‘clusterProfiler’ (v3.16.1) [34]. The enriched GO terms were ranked by adjusted *P* values. Then, we calculated the frequency of FCN1-related GO terms in the top 100 enriched GO terms, which was visualized in a pie chart. Meanwhile, Medical Subject Headings (MeSH) enrichment analysis was performed using the package ‘meshes’ (v1.14.0) [35] based on data source from ‘gene2pubmed’ and ‘C’ (Disease) category.

### Protein–protein interaction (PPI) network construction

Differentially expressed genes identified above were uploaded to the search tool for the retrieval of interacting genes/proteins (STRING) (v11.5) database (https://string-db.org/) [36] for PPI network construction. Then, PPI network visualization was performed using Cytoscape (v3.7.2) [37], where node size depends on degree and edge size depends on combined score. To create a new subnetwork associated with FCN1 in Cytoscape, we first selected FCN1 and then performed the ‘From Selected Nodes, All Edges’ function in the ‘New Network’ function group.

### Cell composition analysis of PIBD mucosal transcriptome data

To characterize the leukocyte composition of mucosal tissue, we downloaded the CIBERSORT [38] R script (v1.03) from ‘https://rdrr.io/github/singha53/amritr/src/R/supportFunc_cibersort.R’, and the leukocyte signature matrix (LM22) from ‘https://github.com/zomithex/CIBERSORT/blob/master/CIBERSORT_data/LM22.csv’. DESeq2-normalized read counts of GSE117993 [27] and normalized microarray data of GSE126124 [28] were taken as the input data for CIBERSORT analysis and *Kruskal–Wallis* test was used to evaluate the difference between two groups. Then, the R packages ‘ggplot2’ was used for visualization.

### Analysis of PIBD single-cell RNA-seq data

Raw expression matrices of mucosal single-cell RNA-seq data from PIBD and control groups were downloaded under the accession number, GSE121380 [29]. Then, the integrated raw data was fed into the established workflow of the R package ‘Seurat’ (v4.0.2) [39]. Following the quality control procedure, cells with 200–5000 detected genes, cells with less than 40000 UMIs and cells with less than 15% UMIs from mitochondrial genes were kept for further analysis. The filtered matrix was processed in Seurat for normalization by using the default method ‘LogNormalize’, followed by dimension reduction. After that, cell clusters were annotated by using both the R package ‘SingleR’ (v1.6.1) [40] with the reference dataset ‘BlueprintEncodeData’ and the known marker genes. The marker genes of the major cell types include *PTPRC* (immune cells), *CD79A* (B cells), *CD3E* (T cells), *CD68* (macrophages), *EPCAM* (epithelial cells), *COL1A1* (fibroblasts) [29]. Next, macrophages with above-average *FCN1* expression were defined as *FCN1*^high^ macrophages, and the rest as *FCN1*^low^ macrophages. To investigate the biological pathway activity in *FCN1*^low^ and *FCN1*^high^ macrophages, hallmark gene sets (v7.5.1) were downloaded from the Molecular Signatures Database (https://www.gsea-msigdb.org/gsea/index.jsp), and then gene set enrichment analysis (GSEA) [41] was performed by using the R package ‘GSEABase’. Intercellular communication network analysis was performed by using the standard workflow of the R package ‘CellChat’ (v1.4.0) [42].

### Human samples

After diagnostic testing, the remaining tissue and blood samples from PIBD patients and non-IBD subjects were collected as our validation cohort. PBMCs were isolated from the whole blood by using Lymphoprep^™^ (1858, Serumwerk Bernburg) according to the manufacturer’s instructions. The protocols were approved by the Ethics Committee of Ruijin Hospital, Shanghai Jiao Tong University School of Medicine.

### Induction of colitis in mice

Three-week-old male C57BL/6 mice were purchased from Phenotek Biotechnology (Shanghai) and housed under specific pathogen-free conditions. Ten mice were randomly assigned to two groups and allowed to acclimate for one week. Then, five mice were administrated by 3% (w/v) dextran sodium sulfate (DSS, MB5535, Meilunbio) solution in their water bottle for 7 consecutive days to induce colitis while 5 control mice received water without DSS. The body weight of each mouse was monitored daily. On the 8th day, the mice were sacrificed for further analysis [43, 44]. All animal experiments were approved by the Ethics Committee of Ruijin Hospital, Shanghai Jiao Tong University School of Medicine.

### Histopathology, immunohistochemistry and immunofluorescence

Samples were collected and immediately fixed in 10% neutral buffered formalin. Paraffin-embedded sections of biopsies were subjected to hematoxylin and eosin (H&E), immunohistochemistry and immunofluorescence staining. Primary antibodies for section staining included: FCN1 (10930-R017, Sino Biological), CD68 (97778 s, CST).

### Quantitative PCR

Total RNA was isolated using RNAex Pro reagent (AG21102, Accurate Biotechnology) and converted to complement DNA using Evo M-MLV. RT Kit (AG11711, Accurate Biotechnology) according to the manufacturer’s instructions. Quantitative PCR was performed using the SYBR® Green Premix Pro Taq HS qPCR Kit (AG11718, Accurate Biotechnology). Gene expression levels were normalized to B2M or Gapdh in human or mouse samples, and evaluated using the 2^−∆∆Ct^ method. The primers used are listed in Additional file 4: Table S3.

### Cell culture and transfection

Human monocyte-like cell line (THP-1) was cultured with RPMI-1640 containing 10% (v/v) fetal bovine serum (FBS) and 1% (v/v) penicillin/streptomycin at 37 °C in a humidified 5% CO_2_ air atmosphere. THP-1 cells were differentiated into M0 macrophages after 48 h exposure to 100 ng/ml PMA (phorbol 12-myristate 13-acetate, S1819, Beyotime), and then polarized towards M1 phenotype by incubation with 100 ng/ml LPS (L2880, Sigma) and 20 ng/ml IFN-γ (11725-HNAS, Sino Biological) or towards M2 phenotype by incubation with 20 ng/ml IL-4 (11846-HNAE, Sino Biological) and 20 ng/ml IL-13 (10369-HNAC, Sino Biological) for 48 h [45]. To determine the effect of FCN1 overexpression in macrophages, THP-1 cells were transfected with pCMV3-FCN1-C-His (HG10930-CH, Sino Biological) or pCMV3-C-His control vectors (CV015, Sino Biological) using Lipofectamine 2000 (11668-019, Invitrogen) in 1.5 ml Eppendorf tubes for 24 h as previously reported [46]. After transfection, THP-1 cells were then transferred into a 12-well plate and cultured with fresh medium containing 100 ng/ml PMA for 24 h. These cells were then cultured with or without 100 ng/ml LPS for 2 h.

### Western blot

Protein was extracted from murine tissues and THP-1-derived macrophages of different phenotypes for western blot. The blots were probed with antibodies against IL-6 (DF6087, Affinity Bioscience), FCN1 (10930-R017, Sino Biological), IL-1β (12242S, CST), GAPDH (GB12002, Servicebio), β-actin (GB12001, Servicebio), caspase-1 (3866S, CST), cleaved caspase-1 (4199S, CST), and NLRP3 (15101S, CST).

### Statistical analysis

Statistical analysis was performed using R software (version 4.0.4). Detailed descriptions of statistical tests are provided in the corresponding bioinformatics methods and Figure Legends.

## Results

### Identification of FCN1 as a candidate biomarker for PIBD diagnosis

**Fig. 1:**
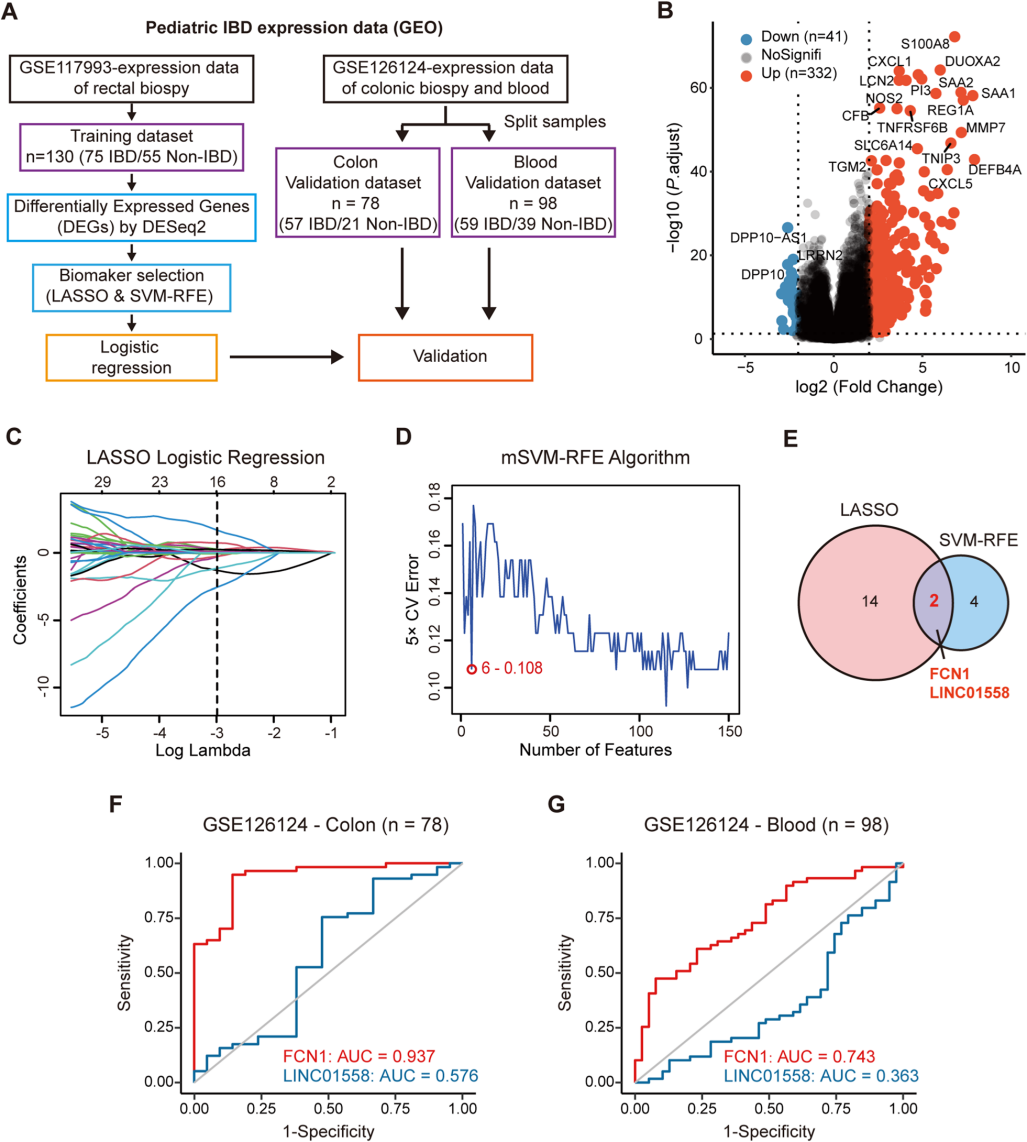
Identification of FCN1 as a candidate biomarker for PIBD diagnosis.** A** Overall workflow for construction and external validation of the diagnostic model. **B** Volcano plot of differentially expressed genes (DEGs) between PIBD and non-IBD rectal biopsy samples of GSE117993. Genes with adjusted *P* < 0.05 and |log2(Fold Change)|> 2 are considered as DEGs, and these DEGs are listed in Table S1. **C** LASSO logistic regression algorithm for biomarker selection. **D** mSVM-RFE algorithm for biomarker selection. **E** Venn diagram visualizing the overlap of selected markers between two algorithms. Receiver operating characteristic (ROC) curves and the corresponding area under the curves (AUCs) of diagnostic prediction models based on FCN1 and LINC01558 in the colon **F** and blood **G** validation datasets (GSE126124)

The mucosal expression profiles of 75 PIBD and 55 non-IBD pediatric patients from the GSE117993 dataset [27] were analyzed to identify disease-specific markers of PIBD. A total of 373 differentially expressed genes (DEGs) between PIBD and non-IBD were subjected to marker selection by using least absolute shrinkage and select options (LASSO) [31] and multiple support vector machine-recursive feature elimination (mSVM-RFE)[32] algorithms (Fig. 1A, B, Additional file 1: Table. S1). The LASSO algorithm identified 16 genes from DEGs as diagnostic markers for PIBD by applying the lambda of minimum mean error; the mSVM-RFE algorithm identified 6 markers as top features of PIBD with a low error rate (Fig. 1C, D). Then, we obtained two overlapping markers, FCN1 and LINC01558, from these two algorithms (Fig. 1E). The diagnostic efficacy of FCN1 and LINC01558 was further validated in an independent PIBD dataset (GSE126124) [28], which consisted of the expression profiles of 78 colon biopsies and 98 peripheral whole blood samples. As shown in Fig. 1F, G, FCN1 showed great diagnostic efficacy in both colon and blood validation sets with area under the curves (AUC) values of 0.937 and 0.743, respectively. In contrast, LINC01558 did not achieve good diagnostic performance in the colon and blood validation datasets (AUCs, 0.576 and 0.363). Taken together, the above results indicate that FCN1 may be a good mucosal and circulating biomarker for the diagnosis of PIBD.

### FCN1 is upregulated in PIBD and associated with immune response

**Fig. 2:**
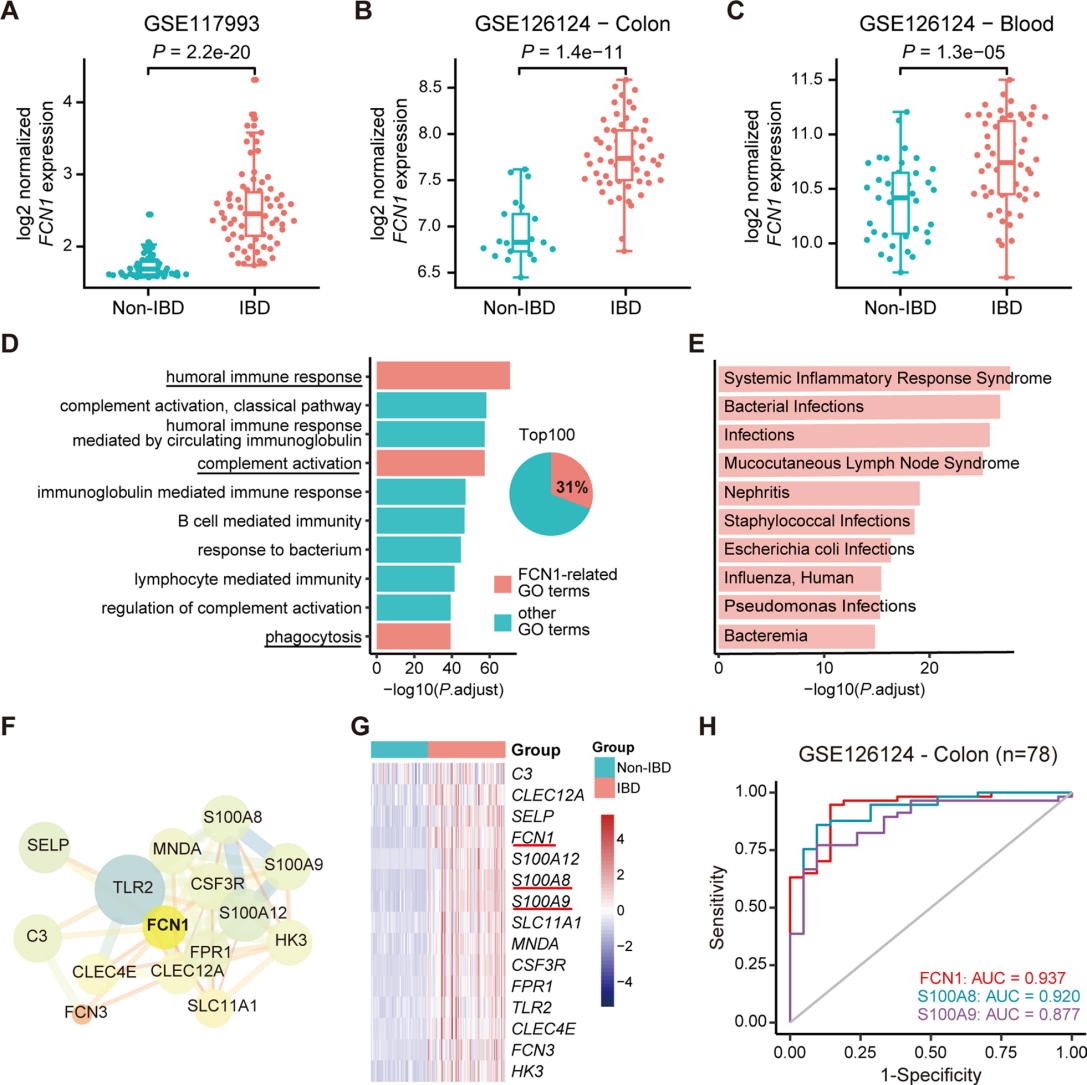
FCN1 is upregulated in PIBD and associated with immune response. **A** The expression level of *FCN1* in the rectal biopsies from PIBD and non-IBD subjects of the GSE117993 RNA-seq dataset. The y axis represents log2-scaled normalized counts by applying the regularized log (rlog) algorithm in DESeq2. Upregulated *FCN1* expression in the colon **B** and peripheral whole blood (**C**) from PIBD and non-IBD subjects of the GSE126124 microarray dataset. The y axis represents log2-scaled normalized gene expression by using the robust multichip average (RMA) algorithm. Unpaired *t* test was employed to calculate *P* values. **D** GO enrichment analysis of DEGs between PIBD and non-IBD subjects. The bar plot exhibits significantly enriched GO terms and the pie chart shows the frequency of FCN1 involved in top 100 enriched GO terms. **E** The bar plot of significantly enriched FCN1-related MeSH terms of DEGs in PIBD compared with non-IBD subjects. **F** The protein-protein interaction subnetwork of DEGs associated with FCN1 based on STRING database. **G** The heatmap visualizing the relative expression of FCN1-associated genes in the GSE117993 dataset. **H** ROC curves and their corresponding AUCs of FCN1, S100A8 and S100A9 (subunits of calprotectin) in the colon validation datasets

The expression level and the underlying role of FCN1 in PIBD remain largely unknown. Using the public RNA-seq dataset (GSE117993), we found that the expression of FCN1 in rectal biopsies from PIBD patients was significantly higher than in non-IBD controls (Fig. 2A). Consistently, in an independent PIBD microarray dataset (GSE126124), we further confirmed that FCN1 expression was increased in both colon biopsies and peripheral whole blood samples from PIBD patients compared with non-IBD subjects (Fig. 2B, C). In addition, DEGs between PIBD and non-IBD identified from the above dataset were subjected to Gene Ontology (GO) analysis and Medical Subject Headings (MeSH) enrichment analysis. The results of GO analysis clearly showed that FCN1 had a high frequency (31%) in the top 100 enriched GO terms. Among the top 10 significantly enriched pathways, FCN1 was involved in humoral immune response, complement activation and phagocytosis (Fig. 2D). The MeSH term enrichment analysis in Fig. 2E showed that FCN1 was closely associated with infection and inflammation. These results suggest that FCN1 is upregulated and associated with immune response in PIBD.

### The mucosal-based diagnostic performance of FCN1 for PIBD is superior to calprotectin

To identify FCN1-related genes from DEGs between PIBD and non-IBD, we performed protein–protein interaction analysis. Among the 14 identified FCN1-related genes (Fig. 2F), S100A8 and S100A9 are the components of calprotectin [47]. Similar to FCN1, the expression of S100A8, S100A9 and other FCN1-related genes were all upregulated in PIBD mucosa compared to non-IBD mucosa (Fig. 2G). Since fecal calprotectin (S100A8/S100A9) is a useful screening biomarker for PIBD diagnosis in clinical practice [12, 13, 16], we compared the diagnostic efficacy of FCN1 with that of S100A8 and S100A9 to determine the potential clinical value of FCN1. In the colon validation set, S100A8 and S100A9 showed efficient diagnostic performance in PIBD, yielding AUCs of 0.920 and 0.877 respectively. Notably, FCN1 demonstrated superior performance for PIBD diagnosis as compared to S100A8 and S100A9 (Fig. 2H). These results further support the potential clinical value of FCN1 in PIBD diagnosis.

### FCN1 expression positively correlates with M0/M1 macrophage infiltration

**Fig. 3:**
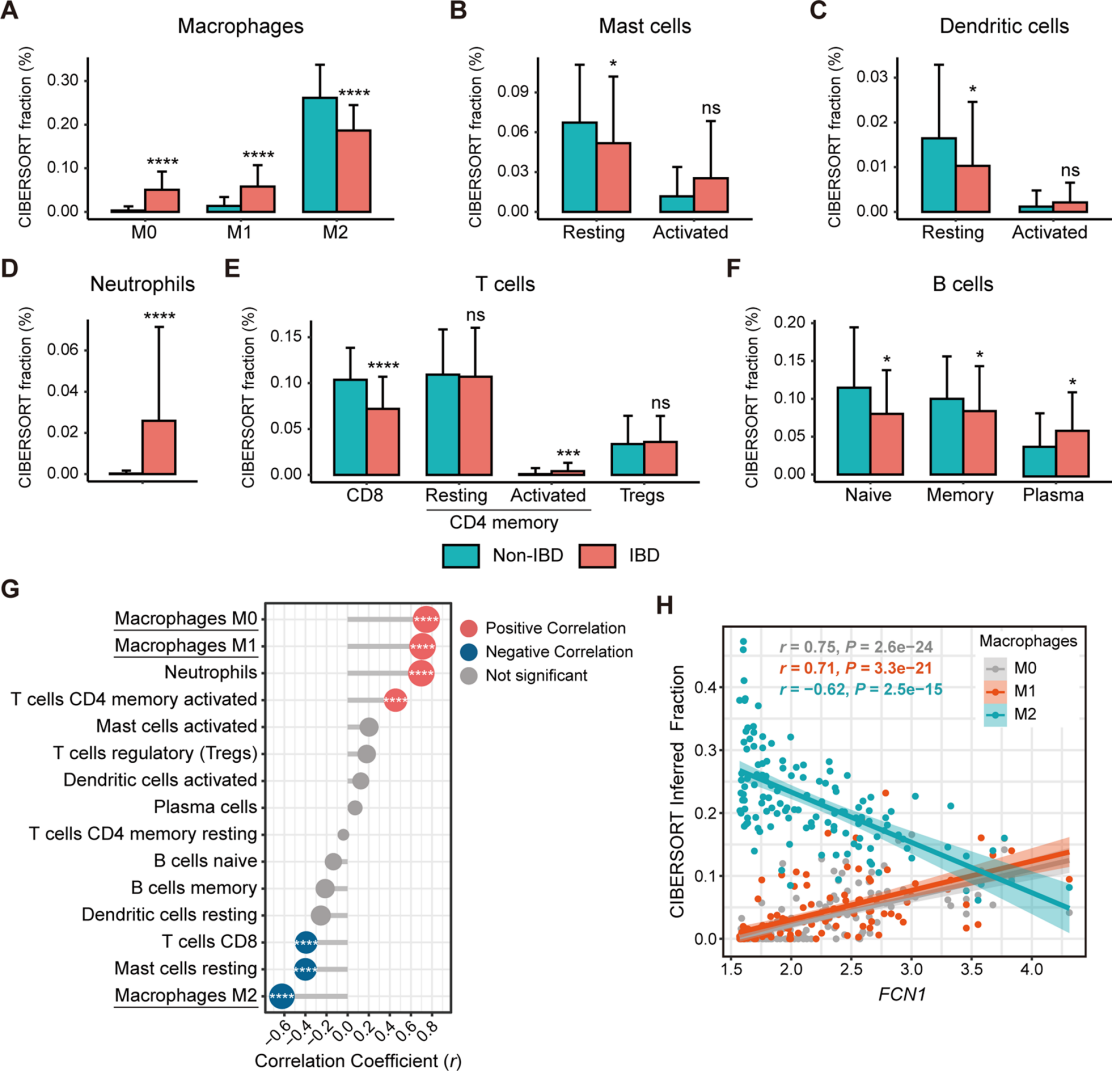
Positive correlation between FCN1 expression and M0/M1 macrophage infiltration. CIBERSORT inferred abundance of macrophages **A**, mast cells **B**, dendritic cells **C**, neutrophils **D**, T cells **E** and B cells **F** in rectal biopsies from PIBD and non-IBD subjects (GSE117993). *Kruskal–Wallis* test was employed to calculate* P* values. *P < 0.05, **P < 0.01, ***P < 0.001, ****P < 0.0001. **G** Spearman correlations between *FCN1* expression and CIBERSORT inferred abundance of infiltrating immune cells. Dot size is proportional to strength of correlation and dot color indicates significance of correlation coefficient (significant positive correlation with Bonferroni corrected *P* < 0.05: red, significant negative correlation: blue, otherwise gray). **** denotes Bonferroni corrected *P* < 0.0001. **H** Scatter plots of the tissue expression of *FCN1* and CIBERSORT inferred abundance of macrophage subsets (M0, M1, M2) in the GSE117993 cohort. The 95% confidence intervals for the correlation between *FCN1* expression and macrophages are (0.66, 0.81) for M0, (0.61, 0.79) for M1, and (-0.72, -0.51) for M2

Immune cells play a critical role in IBD pathogenesis. To identify changes in the immune cell composition of PIBD mucosa, we applied digital cytometry analysis (CIBERSORT) [38] to mucosal expression profiles from the GSE117993 dataset. We found that the immune cell landscape was altered in PIBD rectal mucosa compared to non-IBD controls, including macrophages, mast cells, dendritic cells, neutrophils, T cells and B cells (Fig. 3A–F, Additional file 2: Fig. S1A). Among them, the inferred mucosal abundance of M0 and M1 macrophages was significantly increased, while the abundance of M2 macrophages was decreased in PIBD compared to non-IBD (Fig. 3A). Both resting mast cells and resting dendritic cells were decreased in PIBD, whereas their activated forms were increased, although not reaching statistical significance (Fig. 3B, C). The abundance of infiltrating neutrophils, activated CD4 memory T cells and plasma cells in PIBD were significantly higher than in controls, whereas abundance of CD8 T cells, naïve B cells and memory B cells were lower in PIBD (Fig. 3D–F). Similar changes in immune cell composition were also observed in another PIBD cohort (GSE126124, Additional file 2: Fig. S1B, S2A–F).

To investigate the association between FCN1 expression and immune cell infiltration, we performed Spearman’s correlation analysis. The strongest correlations were found between FCN1 expression and macrophage subsets, including M0, M1 and M2 states (Fig. 3G). As shown in Fig. 3H, FCN1 expression was positively correlated with the CIBERSORT inferred abundance of M0 macrophages (Spearman’s *r* = 0.75, *P* = 2.6 × 10^–24^) and M1 macrophages (Spearman’s *r* = 0.71, *P* = 3.3 × 10^–21^), but negatively correlated with M2-macrophage abundance (Spearman’s *r* = −0.62, *P* = 2.5 × 10^–15^) in the GSE117993 PIBD cohort. These correlations between FCN1 and inferred macrophage subsets were further corroborated in an additional PIBD cohort (GSE126124, Additional file 2: Fig. S2G).

### FCN1 is enriched in macrophages of PIBD colon mucosa and related to hyper-inflammation

**Fig. 4:**
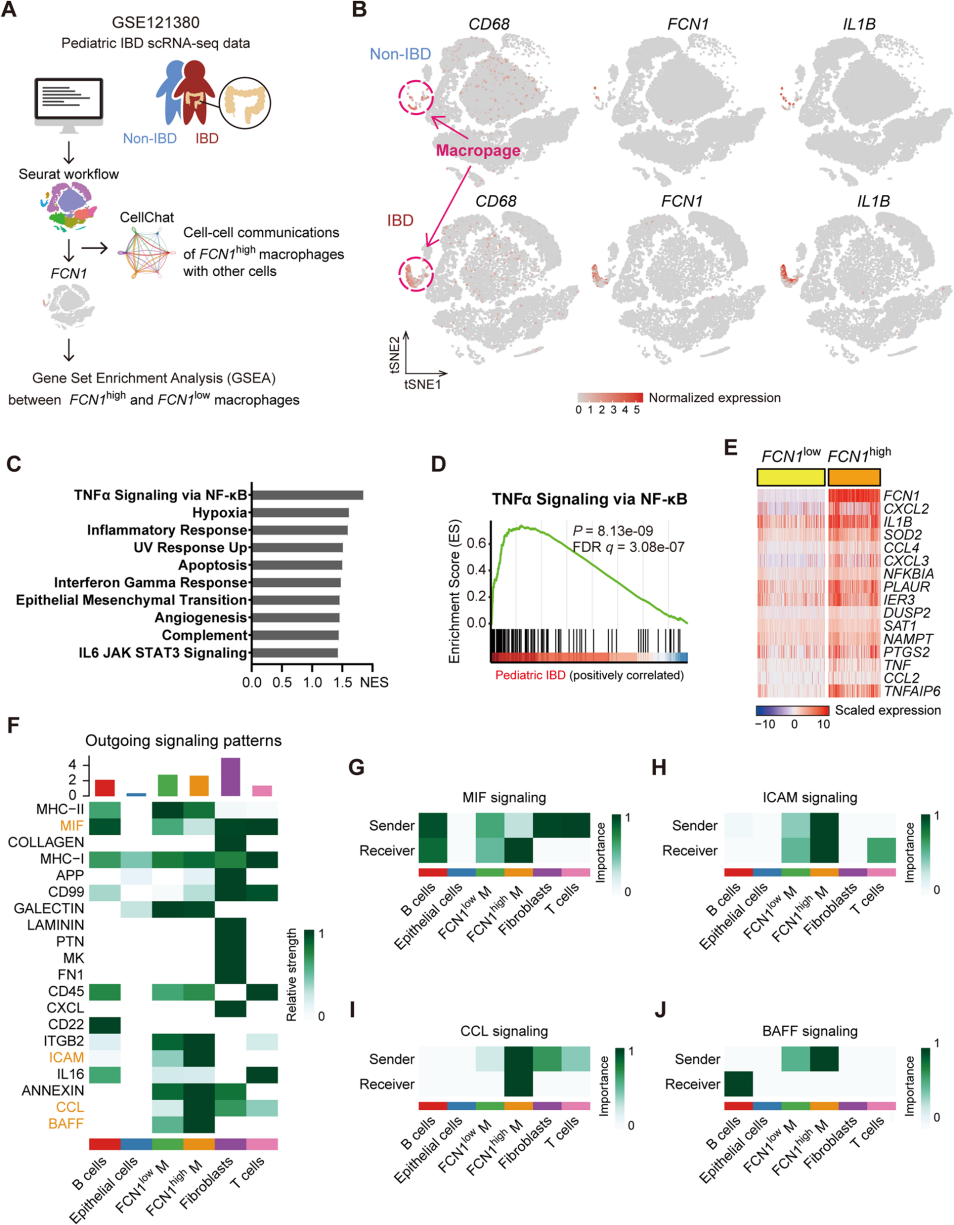
FCN1 is enriched in macrophages of PIBD colon mucosa and related to hyper-inflammation. **A** Diagram depicting the analysis scheme of public single-cell RNA sequencing data (GSE121380). **B** t-SNE plots of *CD68* (macrophage marker), *FCN1* and *IL1B* at the single-cell level. **C** The bar plot of significantly enriched GSEA Hallmark pathways in *FCN1*^high^ macrophages compared with *FCN1*^low^ macrophages. **D** The enrichment plot of TNFα Signaling via NF-κB pathway. **E** Heatmaps of *FCN1* and the top 15 up-regulated genes of TNFα Signaling via NF-κB pathway in *FCN1*^high^ compared with *FCN1*^low^ macrophages. **F** The heatmap showing the overall outgoing signaling patterns of major cell types, including B cells, epithelial cells, *FCN1*^low^ macrophages, *FCN1*^high^ macrophages, fibroblasts, and T cells, as predicted by CellChat. **G-J** Heatmaps of differential signaling networks between *FCN1*^low^ macrophages and *FCN1*^high^ macrophages, including MIF signaling, ICAM signaling, CCL signaling, and BAFF signaling

To further investigate the association between FCN1 expression and macrophage polarization states, we analyzed a single-cell transcriptomic dataset of colon biopsies from non-IBD and PIBD (GSE121380) [29] (Fig. 4A, see Additional file 2: Fig. S3 for cell cluster annotation). *FCN1* was almost expressed in *CD68*^+^ macrophages, and the expression levels of *FCN1* and *IL1B* in macrophages of colon biopsies from PIBD were significantly increased compared to those from non-IBD (Fig. 4B, Additional file 2: Fig. S4). In comparison with *FCN1*^low^ macrophages, several of the top 10 upregulated gene sets in *FCN1*^high^ macrophages were related to hyper-inflammation, such as TNFα signaling via NF-κB, inflammatory response, interferon gamma response, complement and IL-6 signaling (Fig. 4C–E, Additional file 2: Fig. S5, Additional file 3: Table S2). The classical M1 macrophages are generally characterized by pro-inflammatory responses and high expression of TNF, IL-1, IL6, and interferon-induced genes [48–50], suggesting that FCN1^+^ macrophages enriched in PIBD mucosa are similar to pro-inflammatory M1 macrophages.

We then applied CellChat [42] to decipher the intercellular signaling networks in PIBD colon mucosa. Interestingly, we found many differential proinflammatory signaling networks between *FCN1*^low^/*FCN1*^high^ macrophages and other major cell types, such as pathways related to macrophage migration inhibitory factor (MIF), intercellular adhesion molecules (ICAM), CC chemokine ligands (CCL), and B cell activating factor (BAFF) (Fig. 4F). Compared to *FCN1*^low^ macrophages, *FCN1*^high^ macrophages received more signals through the MIF signaling pathway from B cells, fibroblasts, and T cells (Fig. 4G). Notably, *FCN1*^high^ macrophages also exhibited increased autocrine ICAM and CCL signaling relative to *FCN1*^low^ macrophages (Fig. 4H, I). In addition, *FCN1*^high^ macrophages were the major ligand sources of BAFF signaling targeting B cells (Fig. 4J). These observations support that the *FCN1*^high^ macrophages enriched in PIBD colonic mucosa contribute to mucosal inflammation.

### FCN1 demonstrates superior diagnostic performance in the PIBD clinical cohort

**Fig. 5:**
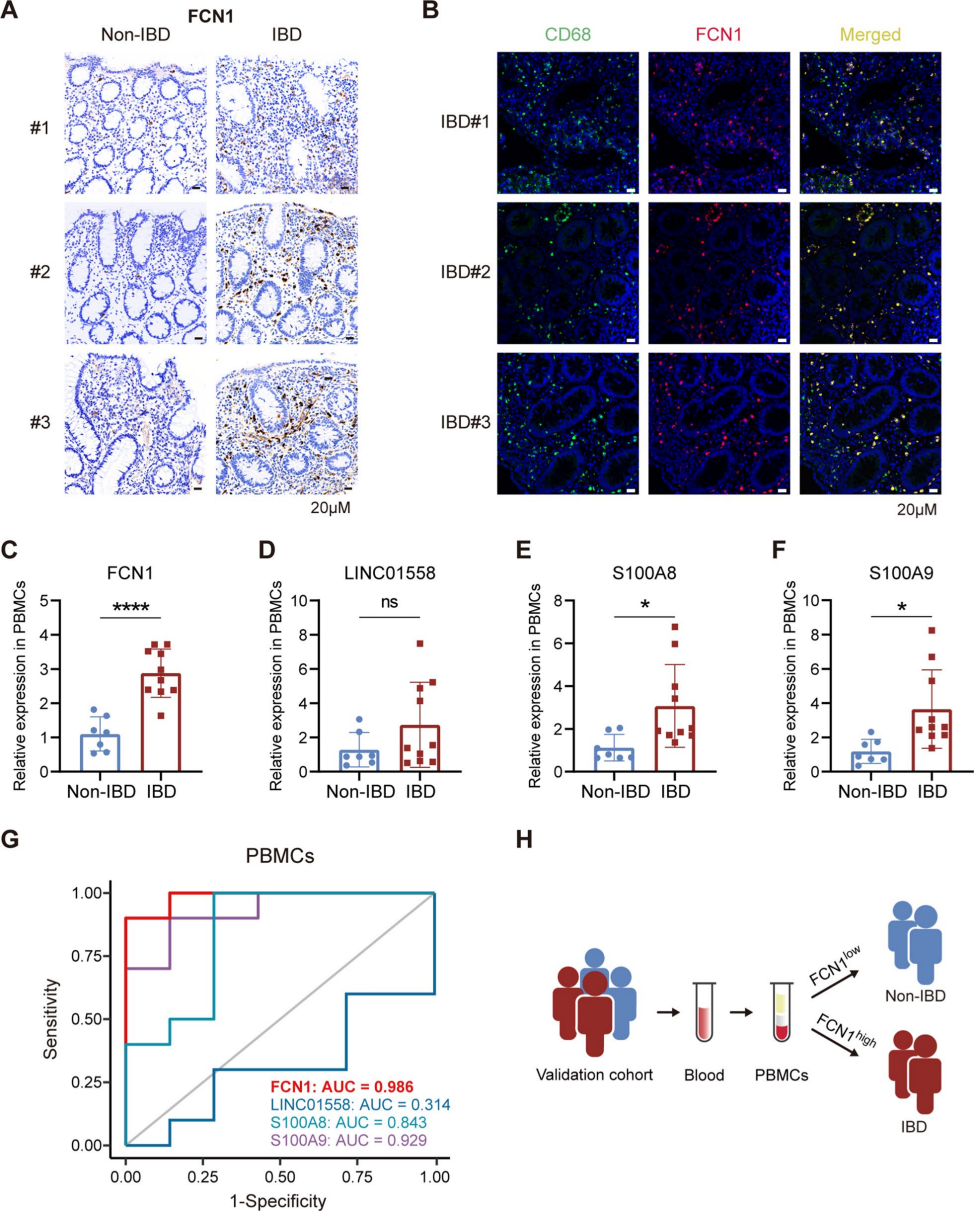
Validation of the diagnostic efficacy of FCN1 in our patient cohort. **A** Immunohistochemistry staining showing increased infiltration of FCN1^+^ cells in colon biopsies of IBD compared with non-IBD subjects. **B** Immunofluorescence staining showing that FCN1^+^ cells (red) colocalize with CD68^+^ cells (green) in the colon biopsies of children with IBD. Yellow indicates double positive cells. **C-F** Relative mRNA expression levels of FCN1, LINC10558, S100A8, and S100A9 in PBMCs from our validation cohort were measured by qRT-PCR. Data represent mean ± SD. Unpaired *t* test was employed to calculate *P* values. *P < 0.05, ****P < 0.0001. **G** ROC curves and their corresponding AUCs of FCN1, LINC01558, S100A8, and S100A9 based on relative mRNA expression levels in PBMCs from our validation cohort. **H** Based on PBMCs isolated from whole blood, FCN1 showed superior sensitivity and specificity for discriminating PIBD patients from non-IBD controls in our validation cohort

Consistent with the findings of the PIBD single-cell transcriptome analysis, the immunostaining results showed that FCN1 expression was remarkably increased in colon biopsies from PIBD patients compared to non-IBD controls (Fig. 5A), and FCN1 was specifically colocalized with CD68^+^ macrophages in PIBD colon biopsies (Fig. 5B). Considering that macrophages in the intestinal mucosa are continuously renewed by circulating monocytes [51], we hypothesized that the isolation of PBMCs from whole blood samples might improve the blood-based diagnostic performance of FCN1, since PBMCs contain a higher percentage of monocytes than whole blood. In our cohort, the mRNA expression levels of FCN1, S100A8, and S100A9 was significantly higher in PBMCs from PIBD patients than from non-IBD controls, but LINC01558 expression was not different between two groups (Fig. 5C–F). As expected, S100A8 and S100A9 showed efficient diagnostic performance in PIBD, whereas LINC01558 did not perform well, yielding AUCs of 0.843, 0.929, and 0.314, respectively. Notably, FCN1 still showed the best sensitivity and specificity to discriminate PIBD patients from non-IBD controls in our validation cohort with the AUC of 0.986 (Fig. 5G). Collectively, these results further indicate that FCN1 is a promising biomarker for PIBD diagnosis (Fig. 5H).

### Expression of FCNB is upregulated in blood, colon, and spleen in murine colitis model

**Fig. 6:**
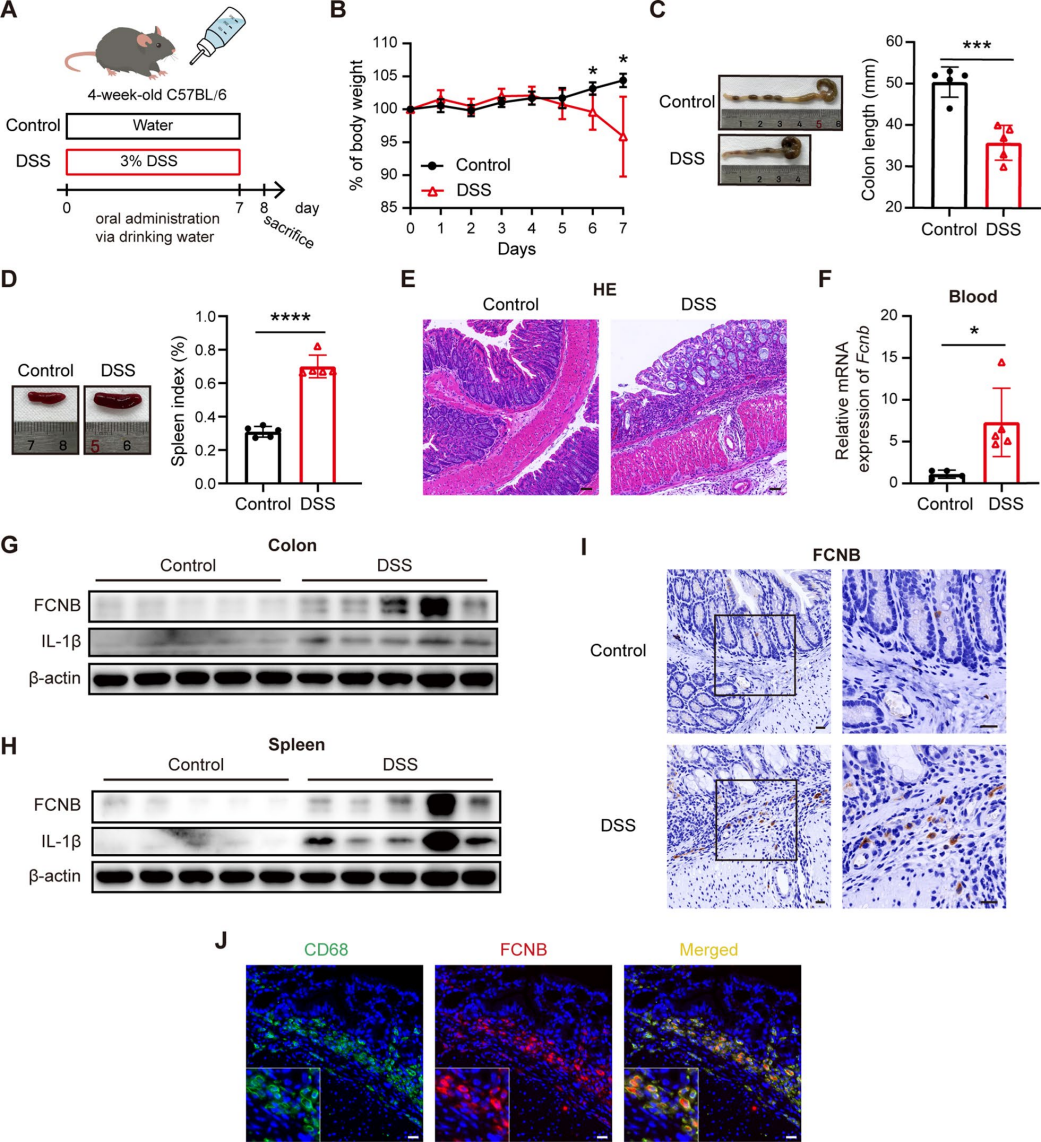
FCNB expression is upregulated and colocalized with CD68 in murine colitis model. **A** Schematic of the animal experiment design. Four-week-old C57BL/6 mice were treated with 3% DSS in drinking water for 7 days to induce colitis, while the control group received water. On the 8th day, mice were sacrificed for further analysis. **B** Mice with DSS-induced colitis showed significantly decreased body weight compared with the control mice. **C** Colon lengths were measured 8 days after DSS treatment. **D** Gross pictures of spleens and spleen index (%). Spleen index (%) is the ratio of spleen weight to body weight. **E** Representative H&E-stained colon tissue sections of control and colitis groups. Scale bars, 50 μM. **F** The mRNA expression levels of *Fcnb* (the murine homologue gene of human *FCN1*) in peripheral whole blood from control and colitis groups were measured by qRT-PCR. The protein expression levels of FCNB and IL-1β in colon **G** and spleen **H** were measured by Western blotting. β-actin was used as a loading control. **I** Immunohistochemistry staining of FCNB in colon of mice. Scale bars, 20 μM. **J** Immunofluorescence staining showing that FCNB^+^ cells (red) colocalize with CD68^+^cells (green) in the colon from mice with DSS-induced colitis. Yellow indicates double positive cells. Scale bars, 20 μM. Data represent mean ± SD. Unpaired *t* test was used to calculate *P* values. *P < 0.05, ***P < 0.001, ****P < 0.0001

Our above results suggest that FCN1 expression was increased in both colorectal mucosa, blood, and PBMCs of PIBD patients. Therefore, we further determined involvement of FCN1 in PIBD using DSS-induced murine colitis model (Fig. 6A). Compared with the control group, four-week-old mice exposed to DSS exhibited loss of body weight, a significant decrease in colon length and enlargement of spleen (Fig. 6B–D). Colitis in DSS-treated mice was ascertained by histopathological analysis of epithelial degeneration and immune cell infiltration (Fig. 6E). Mouse FCNB resembles human FCN1 [18]. Consistent with the findings in human PIBD, the mRNA expression of *Fcnb* was obviously increased in peripheral whole blood of mice with DSS-induced colitis (Fig. 6F), and the protein levels of FCNB were also increased in the colon and spleen tissues of mice with DSS-induced colitis (Fig. 6G, H).

### Increased expression of FCNB is associated with inflammatory macrophage infiltration in the colon mucosa of mice with colitis

Given that FCN1^+^ macrophage infiltration was increased in human PIBD mucosa, and FCN1^high^ macrophages had high expression of IL-1β, we then investigated whether there was an association between FCNB (the murine homologue of human FCN1) and IL-1β in the mouse model of colitis. As expected, the expression of IL-1β was markedly upregulated in colon and spleen tissues from mice with colitis compared to the control group, and tissues with high expression of FCNB also showed higher IL-1β expression (Fig. 6G, H), suggesting the association between FCNB and IL-1β. In addition, increased FCNB^+^ cell infiltration was detected in the colon tissues of mice with DSS-induced colitis when compared to the control group, which was consistent with the findings in human PIBD (Fig. 6I). More importantly, FCNB was specifically colocalized with CD68^+^ macrophages recruited into the colon of mice with colitis (Fig. 6J). Taken together, these results indicate that increased FCNB expression is associated with proinflammatory macrophage infiltration in the colon mucosa of mice with colitis.

### FCN1 promotes caspase-1-mediated IL-1β maturation in macrophages

**Fig. 7:**
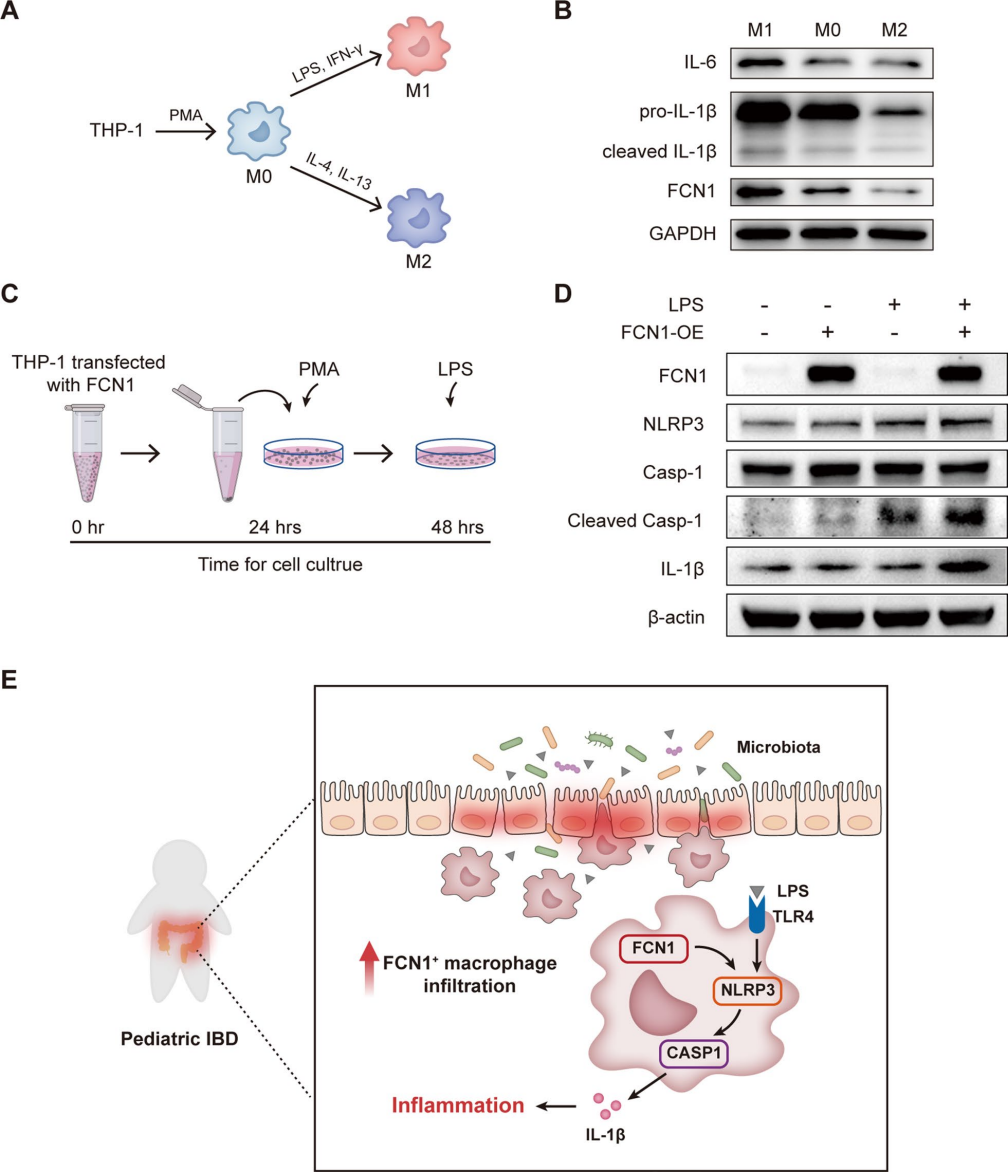
Increased FCN1^+^ macrophage infiltration promotes intestinal inflammation in PIBD. **A** Diagram showing the standardized methods to induce the differentiation of THP-1 cells into M0, M1 and M2 type populations of macrophages. **B** Western blot analysis of M0, M1 and M2 macrophages derived from THP-1 cells. **C** Diagram of transfection and stimulation of THP-1 cells. **D** Western blot analysis of THP-1 cells with different treatments as described in C. FCN1-OE: FCN1-overexpression. **E** The mechanistic diagram of FCN1 function in PIBD. FCN1^+^ macrophage infiltration is increased in the colonic mucosa of PIBD. Impaired intestinal mucosal barrier in PIBD leads to translocation of bacteria and bacterial components such as LPS, and FCN1^+^ macrophages are exposed to LPS. Macrophage FCN1 promotes LPS-induced activation of the proinflammatory cytokine IL-1β via NLRP3-dependent cleavage of caspase-1, resulting in increased intestinal inflammation

To further investigate whether changes in macrophage phenotype are associated with FCN1 expression, THP-1-derived M0, M1, and M2 macrophages were generated (Fig. 7A). Compared with M0 macrophages, M1 polarized macrophages exhibited upregulated expression of FCN1 and pro-inflammatory cytokines, including IL-6, pro-IL-1β, and cleaved-IL-1β, whereas M2 polarized macrophages exhibited downregulated expression of FCN1 and the pro-inflammatory cytokines (Fig. 7B).

The proinflammatory cytokine IL-1β was mainly derived from macrophages and its biological activation typically requires NLRP3 inflammasome-dependent caspase-1 cleavage [52]. Given the observed association between the expression of FCN1 and IL-1β, we questioned whether FCN1 could regulate IL-1β activation. To address this, we overexpressed FCN1 in THP-1-derived macrophages, and found that overexpression of FCN1 slightly increased the protein levels of NLRP3, cleaved caspase-1 and mature IL-1β (Fig. 7C, D). Considering the defective intestinal mucosal barrier and the increased systemic exposure to gut-derived endotoxin (LPS) in patients with PIBD [53, 54], we then exposed THP-1-derived macrophages to LPS. Interestingly, in the presence of LPS, overexpression of FCN1 increased the NLRP3 expression, and greatly upregulated the protein levels of cleaved caspase-1 and mature IL-1β (Fig. 7C, D). Consistently, in the presence of LPS, human recombinant FCN1 protein (rhFCN1) could also increase the protein levels of NLRP3, cleaved caspase-1, and IL-1β (Additional file 2: Fig. S6).

Overall, infiltration of FCN1^+^ macrophages are increased in the colon mucosa of PIBD, and macrophage FCN1 promotes the production of mature IL-1β via NLRP3-dependent cleavage of caspase-1, which contributes to the intestinal inflammation (Fig. 7E).

## Discussion

FCN1 is a member of the soluble pattern recognition molecules that plays an essential role in complement activation through the lectin pathway [55]. Hyperactivation of complement may underlie chronic inflammatory diseases, such as IBD [56]. In this study, we identified FCN1 as a novel promising mucosal and circulating biomarker, which could accurately discriminate PIBD patients from non-IBD children. Notably, FCN1 showed great blood-based performance for PIBD discrimination in our clinical validation cohort, with the AUC of 0.986. The expression of FCN1 was significantly increased in both colorectal biopsies and blood of PIBD compared to non-IBD in both public bulk transcriptomic datasets and our clinical cohort, which was further validated in the mouse model of DSS-induced colitis.

We also observed the association of FCN1 with S100A8 and S100A9, which are the components of calprotectin [47]. Fecal calprotectin (S100A8/S100A9), leaking from inflamed mucosa of IBD, has been the most recorded biomarker of intestinal inflammation [12, 13], and is useful for PIBD diagnosis in clinical practice [14, 16]. Our results highlighted that the mucosal and blood-based diagnostic performance of FCN1 in IBD was superior to that of S100A8 and S100A9, further supporting the potential clinical value of FCN1 in PIBD diagnosis. It is worth further exploring whether fecal FCN1 could serve as a diagnostic biomarker for PIBD, as elevated mucosal FCN1 may leak into the lumen together with calprotectin and be subsequently excreted in the feces.

Dysfunction of the innate and adaptive immune systems is considered as an important pathogenetic factor of IBD. For instance, the expansion of IgG^+^ plasma cells with reduced diversity and maturation [57], as well as increased activated Th17 cells but decreased CD8^+^ T cells, γδ T cells and Treg cells [58] were detected in inflamed IBD mucosa, which might exacerbate inflammation; additionally, the myeloid cell populations such as altered immature macrophages were accumulated in the inflamed colon of IBD patients, where these cells produced excessive inflammatory cytokines and aggravated epithelial damage [22].

In our study, we demonstrated the altered immune cell landscape in the PIBD mucosa, which is consistent with the findings of previous work. Among them, the inferred mucosal abundance of M0/M1 macrophages is significantly increased in PIBD compared to non-IBD individuals, and the upregulation of FCN1 correlates with the increase of M0/M1 macrophages based on the bulk transcriptomes. We also found high FCN1 expression in M1-like macrophage populations in the PIBD single-cell transcriptomic data and confirmed the expansion of FCN1^+^ macrophages in both PIBD patients and mouse models. The high expression level of FCN1 in M1 macrophages suggests that it may play an additional role in the inflammatory response.

In addition to complement activation, FCN1 could dock onto the transmembrane G protein-coupled receptor 43 to sense pathogens and trigger intracellular signaling to regulate host defense [59]. It has also been reported that FCN1 binds to specific lymphocyte subsets such as activated T cells, via sialic acids on the cell surface [19]. However, far too little attention has been paid to the detailed mechanisms of FCN1 in autoimmune and autoinflammatory diseases in the past few decades [20]. Most previous studies have only focused on the aberrant expression of FCN1 in some autoimmune diseases [60–62] and its correlation with disease activity [63–65].

In our study, we found the association between the expression of FCN1 and IL-1β both in vivo and in vitro, indicating that FCN1 may be involved in the maturation of IL-1β. IL-1β is a pro-inflammatory cytokine that plays a critical role in inflammatory disorders [66] and is typically regulated by the NLRP3-caspase-1 axis [52]. Our findings suggest that upregulated FCN1 expression in macrophages slightly promoted the expression of NLRP3, cleavage of caspase-1 and subsequent activation of IL-1β. Impaired intestinal mucosal barrier in PIBD leads to increased systemic exposure to gut microbiota-derived LPS [53, 54]. We observed that in the presence of LPS, overexpression of FCN1 in macrophages greatly enhanced caspase-1 cleavage, resulting in significantly increased production of mature IL-1β, which promotes the intestinal inflammation. It was previously reported that anti-FCN1 monoclonal antibody could alleviate experimental arthritis in the mouse model [67]. Similarly, targeting FCN1^+^ macrophages may help alleviate the intestinal inflammation in PIBD patients. Future studies are needed to investigate precisely how FCN1 facilitates LPS-induced IL-1β activation via the NLRP3-caspase-1 axis, and the potential of FCN1 as a therapeutic target for IBD.

## Conclusions

In conclusion, our study has demonstrated that FCN1 is a novel promising mucosal and circulating biomarker for PIBD diagnosis. Macrophages expressing FCN1, which are enriched in PIBD mucosa, exhibit proinflammatory phenotypes. Moreover, FCN1 greatly promotes LPS-induced activation of the proinflammatory cytokine IL-1β via NLRP3-dependent cleavage of caspase-1 in macrophages. Therefore, targeting FCN1^+^ macrophages may help alleviate the intestinal inflammation in PIBD patients.

## Supplementary Information

**Additional file 1: Table S1.** Differentially expressed genes of GSE117993.

**Additional file 2: Figure S1.** Immune infiltration landscape in PIBD mucosa with CIBERSORT. **Figure S2.** Positive correlation between FCN1 expression and M0/M1 macrophage infiltration in the GSE126124 dataset. **Figure S3.** Cell type analysis of single-cell RNA-seq data (GSE121380). **Figure S4.** Upregulated expression of *FCN1* and *IL1B* in mucosal macrophages from PIBD subjects of the single-cell RNA-seq dataset (GSE121380). **Figure S5.** The GSEA enrichment plots of significantly enriched pathways in *FCN1*^high^ macrophages compared to *FCN1*^low^ macrophages in Fig. 4C. **Figure S6.** Effect of human recombinant FCN1 protein (rhFCN1) on the NLRP3/IL-1β axis was investigated in THP-1-derived macrophages.

**Additional file 3: Table S2.** GSEA results for *FCN1*^high^ and *FCN1*^low^ macrophages.

**Additional file 4: Table S3.** qRT-PCR primer sequences.

## Data Availability

Publicly available datasets analyzed in this study were downloaded from GEO database (https://www.ncbi.nlm.nih.gov/geo/) under the accession numbers: GSE117993, GSE126124, GSE121380.

## Abbreviations

FCN1: Ficolin-1
FCNB: Ficolin-B
IBD: Inflammatory bowel diseases
PIBD: Pediatric inflammatory bowel diseases
PBMCs: Peripheral blood mononuclear cells
RNA-seq: RNA-sequencing
GEO: Gene expression omnibus
DEGs: Differentially expressed genes
LASSO: Least absolute shrinkage and select options
mSVM-RFE: Multiple support vector machine-recursive feature elimination
AUC: Area under the ROC curve
GO: Gene ontology
MeSH: Medical subject headings
PPI: Protein-protein interaction
IL1B: Interleukin 1β
GSEA: Gene set enrichment analysis
H&E: Hematoxylin and eosin
IHC: Immunohistochemistry
DSS: Dextran sulfate sodium
MIF: Macrophage migration inhibitory factor
ICAM: Intercellular adhesion molecules
CCL: CC chemokine ligands
BAFF: B cell activating factor
Casp-1: Caspase-1
rhFCN1: Human recombinant FCN1 protein
