# Supplementary material for: Identification of FCN1 as a novel macrophage infiltration-associated biomarker for diagnosis of pediatric inflammatory bowel diseases
Source: J Transl Med. 2023 Mar 17;21:203. doi: 10.1186/s12967-023-04038-1 (PMC10022188; doi:10.1186/s12967-023-04038-1)
Supplement: Supplementary file 2 — Additional file 2: Figure S1. Immune infiltration landscape in PIBD mucosa with CIBERSORT. Figure S2. Positive correlation between FCN1 expression and M0/M1 macrophage infiltration in the GSE126124 dataset. Figure S3. Cell type analysis of single-cell RNA-seq data (GSE121380). Figure S4. Upregulated expression of FCN1 and IL1B in mucosal macrophages from PIBD subjects of the single-cell RNA-seq dataset (GSE121380). Figure S5. The GSEA enrichment plots of significantly enriched pathways in FCN1high macrophages compared to FCN1low macrophages in Fig. 4C. Figure S6. Effect of human recombinant FCN1 protein (rhFCN1) on the NLRP3/IL-1β axis was investigated in THP-1-derived macrophages. [file 12967_2023_4038_MOESM2_ESM.docx]

**
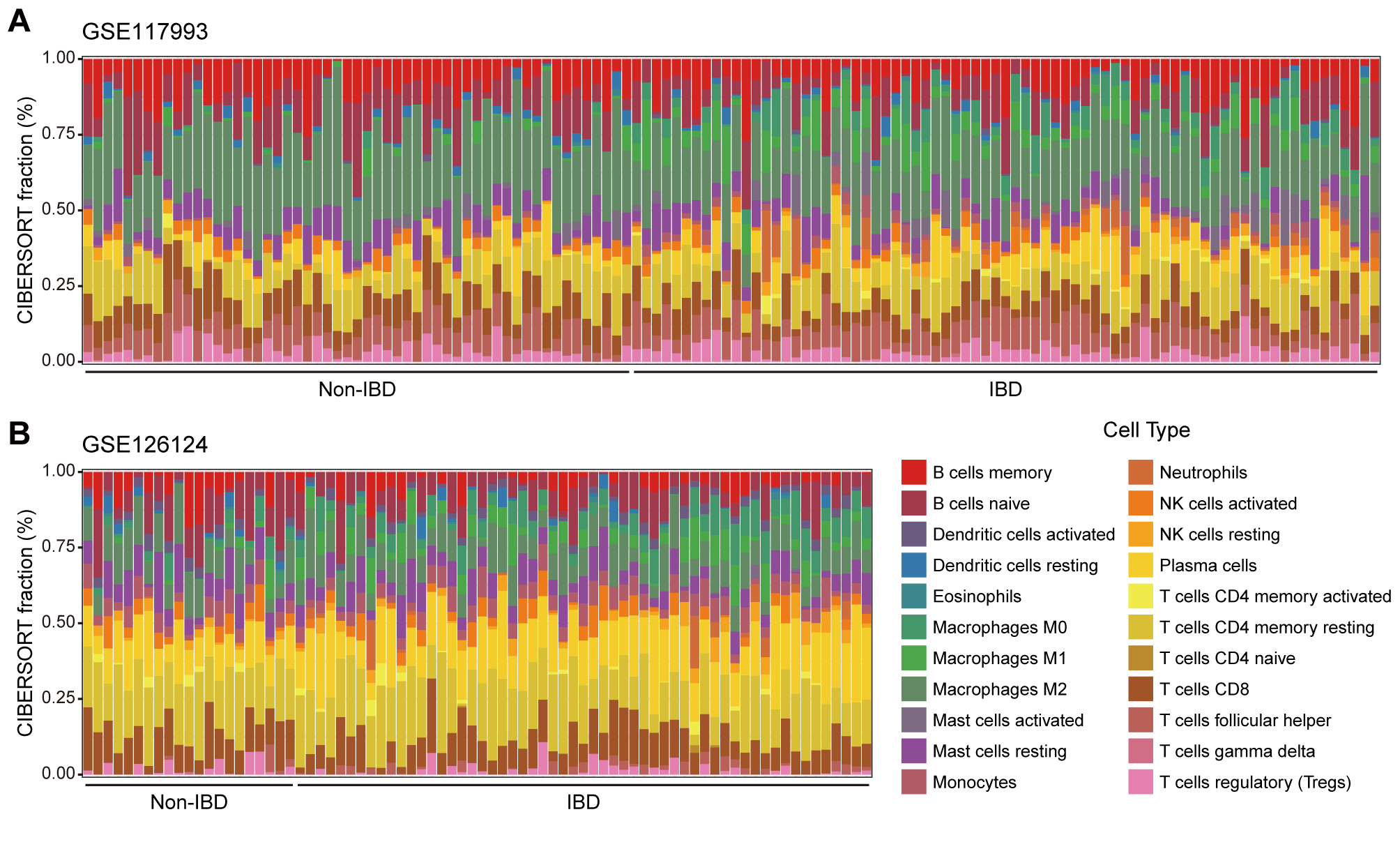
**

**Fig. S1** Immune infiltration landscape in PIBD mucosa with CIBERSORT. **A** The inferred proportion of 22 cell types in the rectal mucosa of non-IBD and PIBD shown in bar plots (GSE117993). **B** The inferred proportion of 22 cell types in the colon mucosa of non-IBD and PIBD (GSE126124)


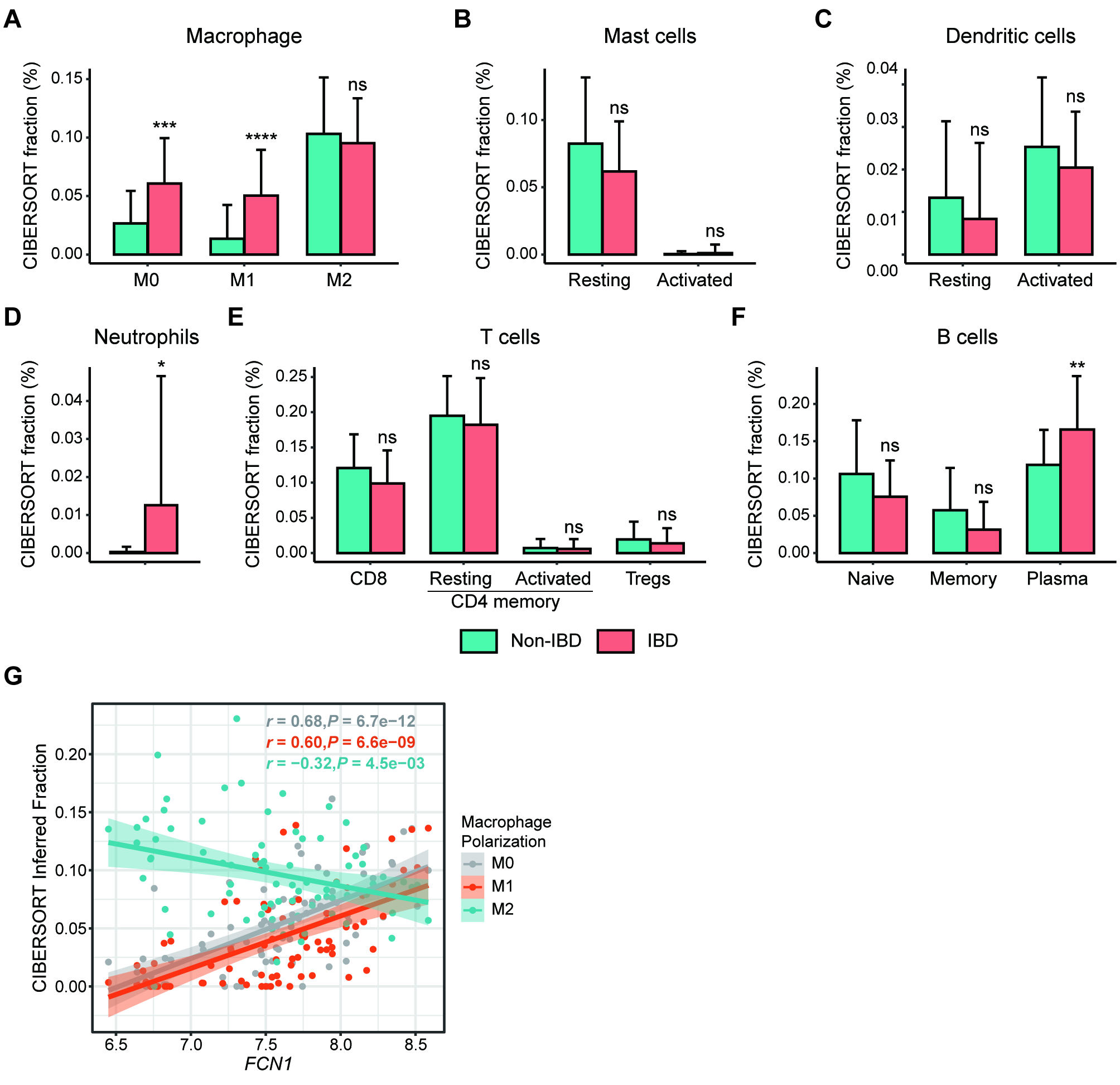


**Fig. S2** Positive correlation between FCN1 expression and M0/M1 macrophage infiltration in the GSE126124 dataset. CIBERSORT inferred abundance of macrophages (**A**), mast cells (**B**), dendritic cells (**C**), neutrophils (**D**), T cells (**E**) and B cells (**F**) in colon biopsies from PIBD and non-IBD subjects (GSE126124). *Kruskal-Wallis* test was employed to calculate *P* values. **P* < 0.05, ***P* < 0.01, ****P* < 0.001, *****P* < 0.0001. **G** Scatter plots of *FCN1* expression and CIBERSORT inferred abundance of macrophage subsets (M0, M1, M2) in the GSE126124 cohort.

**
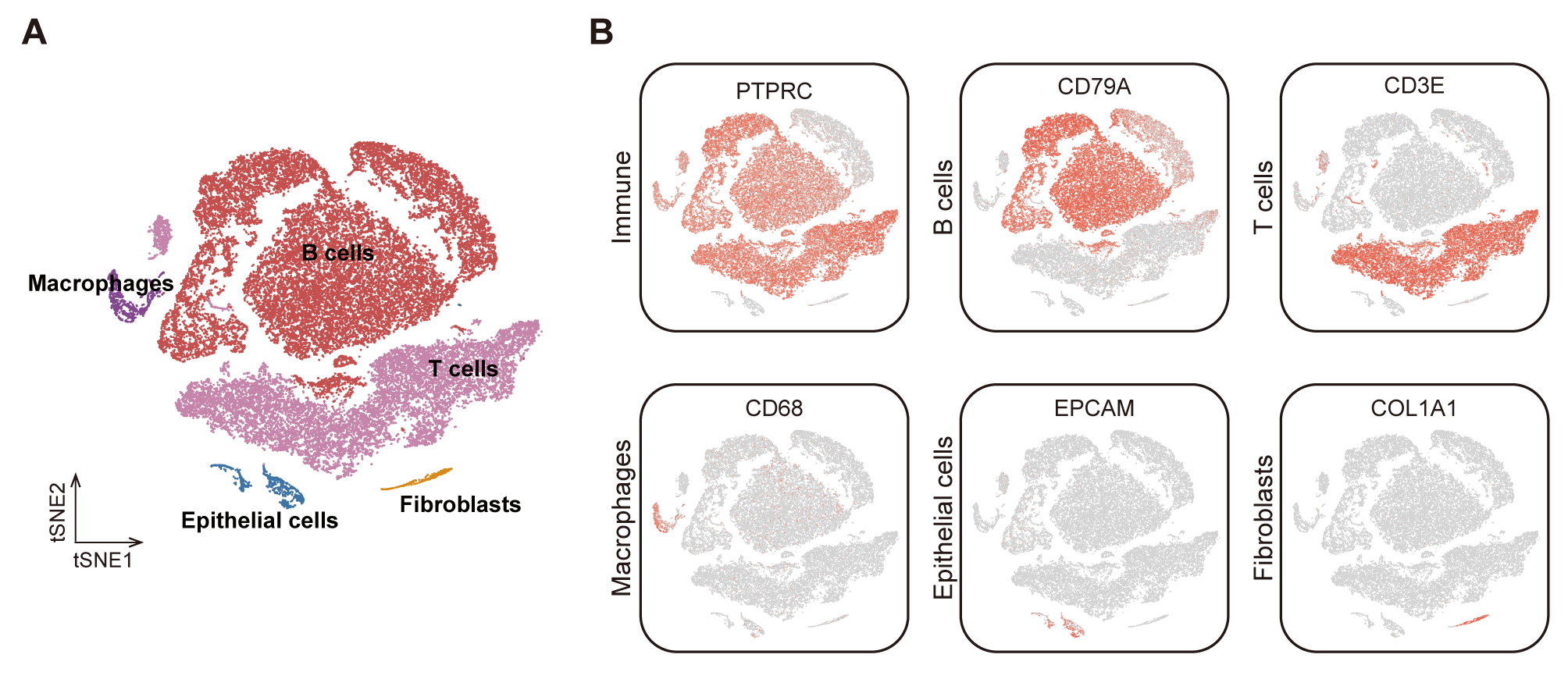
****Fig. S3** Cell type analysis of single-cell RNA sequencing data (GSE121380). **A** The tSNE plot of single-cell RNA sequencing data, showing 5 clusters in the plot. Cell clusters were shown in different colors. **B** tSNE plots showing gene expression of known markers in indicated cell clusters.

**
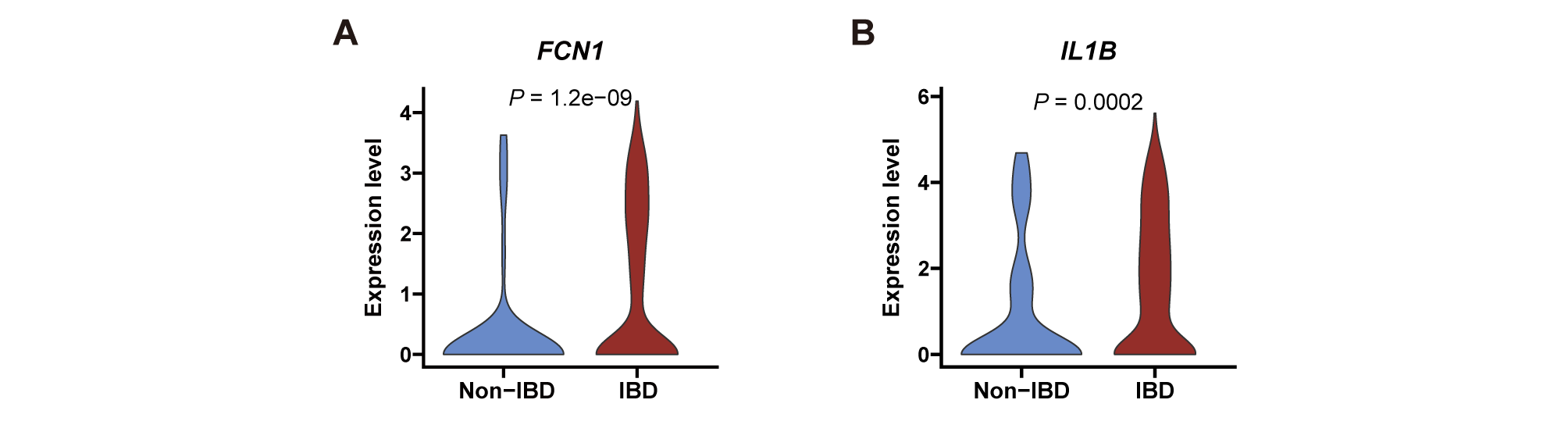
**

**Fig. S4** Upregulated expression of *FCN1* and *IL1B* in mucosal macrophages from PIBD subjects of the single-cell RNA-seq dataset (GSE121380). Violin plots showing the expression levels of *FCN1* (**A**) and *IL1B* (**B**) in mucosal macrophages of PIBD patients.

**
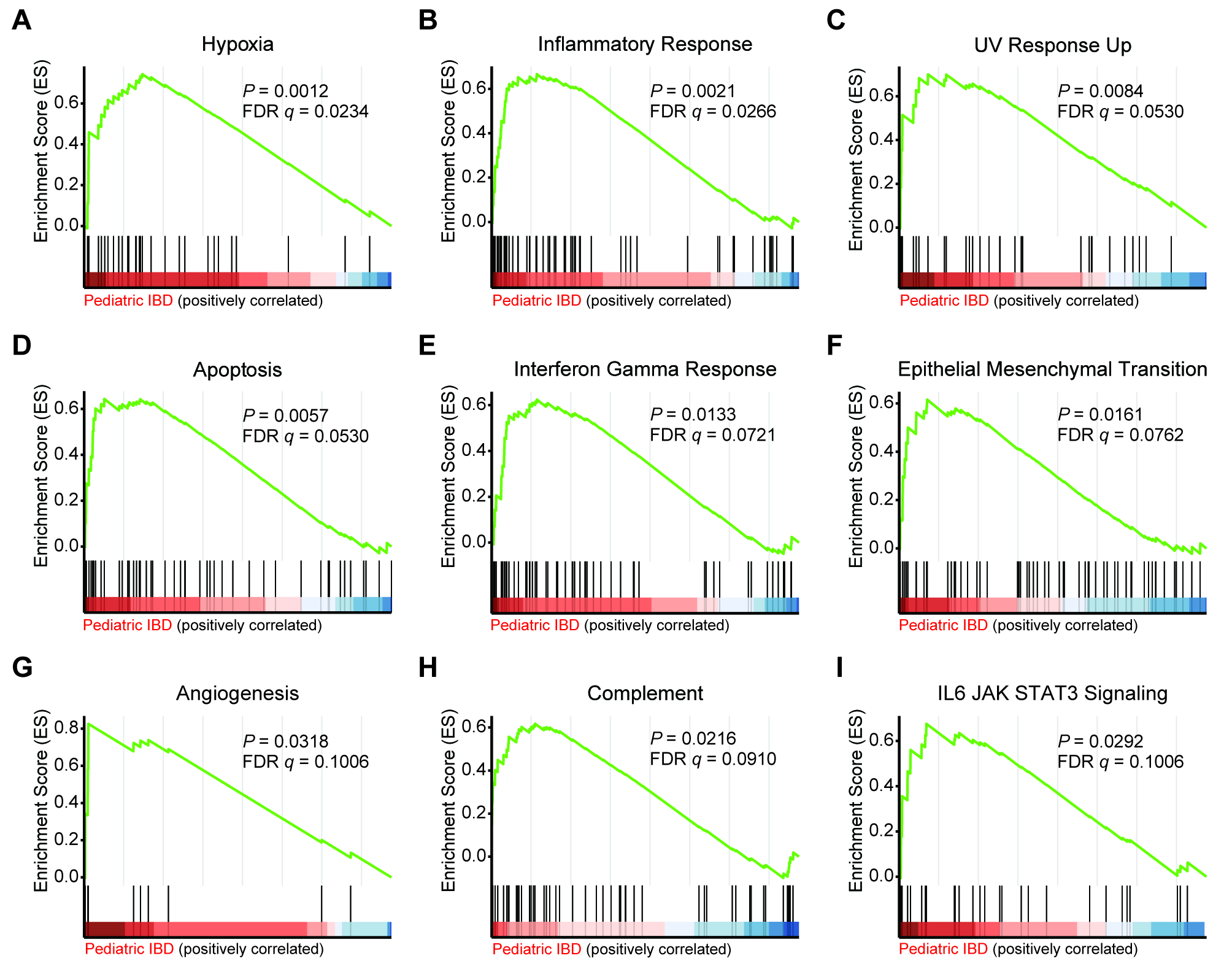
**

**Fig. S5 A-H** The GSEA enrichment plots of significantly enriched pathways in *FCN1*^high^ macrophages compared with *FCN1*^low^ macrophages in Fig. 4C.


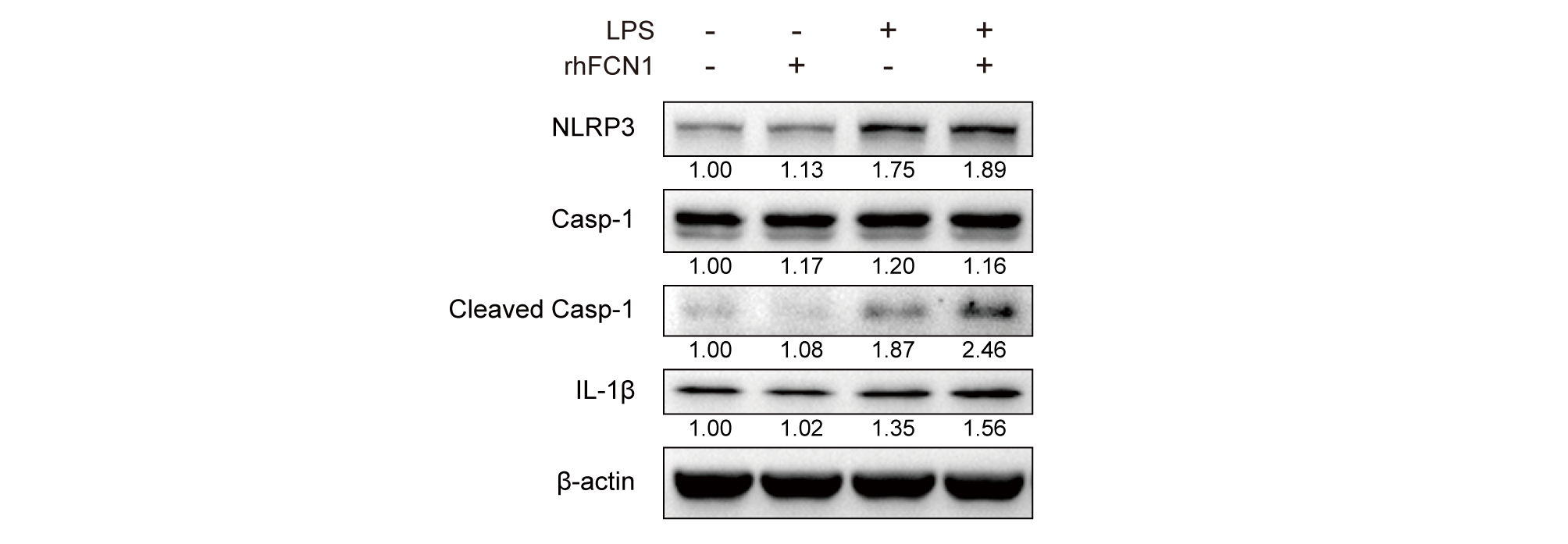


**Fig. S6** Effect of human recombinant FCN1 protein (rhFCN1) on the NLRP3/IL-1β axis was investigated in THP-1-derived macrophages. THP-1 cells were treated with 100 ng/ml PMA for 24 hours, and then incubated with 20ng/ml rhFCN1 (10930-H08H, Sino Biological); after incubation for 24 hours, these cells were cultured with or without 100 ng/ml LPS for another 2 hours. The numbers below the bands indicate the expression levels relative to the control after normalization against β-actin.
