# Supplementary material for: Identification of FCN1 as a novel macrophage infiltration-associated biomarker for diagnosis of pediatric inflammatory bowel diseases
Source: J Transl Med. 2023 Mar 17;21:203. doi: 10.1186/s12967-023-04038-1 (PMC10022188; doi:10.1186/s12967-023-04038-1)
Supplement: Supplementary file 4 — Additional file 4: Table S3. qRT-PCR primer sequences. [file 12967_2023_4038_MOESM4_ESM.docx]

**Table S3** qRT-PCR primer sequences

| **Gene** | **Forward Primer 5’-3’** | **Reverse Primer 5’-3’** |
| --- | --- | --- |
| FCN1 | ATTCAAGGTGGCTGACGAGG | GAAGTTGTTGTTGTGGCCCG |
| LINC01558 | AGCTGGAGATGTGGTCAACG | ATGGAGCCTTCCCAGTGTTG |
| S100A8 | ATGTCTCTTGTCAGCTGTCTTTCA | GGAGTACTTGTGGTAGACGTCG |
| S100A9 | ACCAATACTCTGTGAAGCTGGG | CCTCCATGATGTGTTCTATGACCTT |
| B2M | AGATGAGTATGCCTGCCGTG | TCATCCAATCCAAATGCGGC |
| Fcnb | ACAAGCAGCTCACCATCCTTC | GTAGCACATGACTGAGAGGGC |
| Gapdh | GCATTGTGGAAGGGCTCATG | TTGCTGTTGAAGTCGCAGGAG |
